# Effect of Biomolecules on the Nanostructure and Nanomechanical Property of Calcium-Silicate-Hydrate

**DOI:** 10.1038/s41598-018-27746-x

**Published:** 2018-06-22

**Authors:** Mahsa Kamali, Ali Ghahremaninezhad

**Affiliations:** Department of Civil, Architectural and Environmental Engineering, University of Miami, Coral Gables, FL 33146 United States

## Abstract

Inspired by nature, this paper investigates the effect of biomolecules, such as amino acids and proteins, on the nanostructure and mechanical stiffness of calcium-silicate-hydrate (C-S-H). Amino acids with distinct functional groups, and proteins with different structures and compositions were used in the synthesis of the C-S-H nanocomposite. The atomic structure was examined using X-ray diffraction (XRD) and Fourier transform infrared spectroscopy (FTIR). The morphology was investigated using scanning electron microscopy (SEM) and atomic force microscopy (AFM). AFM nanoindentation was used to evaluate the Young’s modulus of the modified C-S-H. Positively charged, H-bond forming and hydrophobic amino acids were shown to influence the atomic structure of C-S-H. The effect of negatively charged amino acid on atomic structure was more pronounced at higher C/S ratio. A noticeable increase in silicate polymerization of C-S-H modified with proteins at high C/S ratio was observed. The microscopic examination demonstrated a globular morphology for all samples except for C-S-H modified with hemoglobin, which showed a platelet morphology. The Young’s modulus of C-S-H with amino acids and proteins showed a general reduction compared to that of the control C-S-H.

## Introduction

Certain biological composites, such as bones, teeth, and nacre of abalone shells, exhibit superior mechanical and physical performance unmatched by engineering materials^1–3^. This is attributed to the interaction between organic and inorganic materials governing the microstructure of these materials. Inspired by nature, there have been intense research efforts to understand the underlying mechanisms for superior mechanical and physical properties of biological nanocomposites^1,4–6^. A major goal of these studies is to enable synthesis of materials with improved mechanical and functional properties exceeding those of traditional engineering materials.

Concrete is the most widely used infrastructure material and its production is expected to increase at a rate of 5% annually^7^. Calcium silicate hydrate (C-S-H) is the primary hydration product of cement and water and thus, is considered the main contributor to the mechanical properties and durability of cementitious materials^8–11^. In the cement chemistry nomenclature C=CaO, S=SiO_2_, and H=H_2_O.

There is an increasing interest in innovative approaches to manipulate the structure of C-S-H at the nanoscale as a way to improve macroscale properties^12–20^. Inspired by nature, the incorporation of polymers as an organic compound in C-S-H is considered to be promising to achieve enhanced mechanical performance in C-S-H. Matsuyama *et al*.^13,17^ were the first to investigate the possibility of intercalation of polymers with different charges into the structure of C-S-H with various calcium to silicate ratios. Although they did not report a successful intercalation of small sized polymer molecules, they claimed that polymers with higher molecular size can intercalate between the C-S-H layers. Based on their findings, the intercalation of cationic polymers between C-S-H layers are more favorable at low C/S values. However, anionic polymers are more likely to intercalate at high C/S. They also reported a possible intercalation of non-ionic poly(vinyl alcohol) which was related to the interaction of the polymer with C-S-H, facilitated through the partial ionization of the polymer molecule at the high pH of the solution. Pelisser *et al*.^18^ claimed that a lower molecular weight poly(vinyl alcohol) has the potential to intercalate between the C-S-H layers. In another study, Pelisser *et al*.^19^ investigated the interaction of poly(diallyldimethylammonium chloride) with C-S-H. According to their findings, at C/S of 0.8, some poly(diallyldimethylammonium chloride) intercalated between the C-S-H layers. However, the rest of the polymer was adsorbed on the surface of C-S-H and decreased the mechanical properties of the C-S-H complex by decreasing its packing density. Alizadeh *et al*.^15^ synthesized C-S-H with aniline monomers and then polymerized the monomers to achieve C-S-H/polyaniline nanostructures. They reported a potential increase in the silicate polymerization at various C/S levels in the polyaniline modified C-S-H. Khoshnazar *et al*.^16^ investigated the effect of low molecular size nitrobenzoic acid on the structure of C-S-H and reported a possible intercalation at a low polymer concentration. Minet *et al*.^21^ showed that small molecular sized organic molecules are able to intercalate between the C-S-H layers without changing the structural framework of C-S-H. However, phase separation occurred for the C-S-H/polymer complexes when the polymer molecule was large in size or highly hydrophobic. In another study^22^, they reported a successful synthesis of C-S-H with polymers directly bonded to its structure via covalent bonds.

Although the effect of cationic and anionic polymers on the structure of C-S-H has been studied by researchers, the effect of biomolecules has not been investigated. Biomolecules are known to play a crucial role in the growth and microstructure formation of some biological nanocomposites that exhibit superior mechanical properties^1,23–25^. Thus, biomolecules are considered promising compounds to manipulate the structure of bio-inspired materials^26–29^. The complex structure and chemical functionalities of biomolecules can permit a wide range of interactions with C-S-H. The ability to modify the characteristics of C-S-H through a specific combination of interactions with biomolecules provides a pathway to manipulate the structure, and the physical and mechanical properties of C-S-H. In a recent study, Picker *et al*.^30^ studied the adsorption of peptides on C-S-H with different calcium to silicate ratios to shed light on the possible interactions occurring between C-S-H and peptides. The outcomes of their study revealed the role of various functional groups of peptides on interaction with C-S-H. They showed that an organic additive should contain all three charged, H-forming and hydrophobic functional groups in order to adsorb strongly on C-S-H. This suggests that biomolecules with an ability to interact with other molecules through different functional groups can be considered a promising organic compound to manipulate the properties of C-S-H/biomolecule nanocomposite systems. Motivated by their promise in controlling the nucleation, growth, and self-assembly of inorganic systems^30,31^, this paper, for the first time, aims to investigate the effect of biomolecules on the structure and mechanical properties of C-S-H. Amino acids include both amine and carboxyl functional groups, as well as a side chain (except glycine), in their molecular structure^32^. A chain of amino acids build a peptide, which is the building block of proteins. Amino acids based on the functional group of their side chain are classified into three different categories of charged, polar (hydrogen bonding) and non-polar (hydrophobic)^32^.

In order to elucidate the effect of biomolecules on C-S-H, four amino acids with different functional groups and three proteins with different compositions are used to synthesize C-S-H/biomolecule nanocomposites. Characterization methods, including XRD and FTIR, were used to study the structure of the nanocomposites. Morphology examination was performed using SEM and AFM, and the Young’s modulus was elucidated using nanoindentation.

In this study, the synthesis of the nanocomposites is through the direct precipitation method as detailed in the next section. In real applications for cementitious materials, we envision use of biomolecules in the form of chemical admixtures. Chemical admixtures are widely used to improve the physical and chemical properties of cementitious materials. It should be noted that use of biomolecules as chemical admixtures in cementitious materials requires further investigations to elucidate the interaction between biomolecules and other phases present in the microstructure of cementitious materials. This study is focused on C-S-H as the main binding phase of cementitious materials and lays the foundation for the future investigations.

## Experiments

### Materials

**Figure 1:**
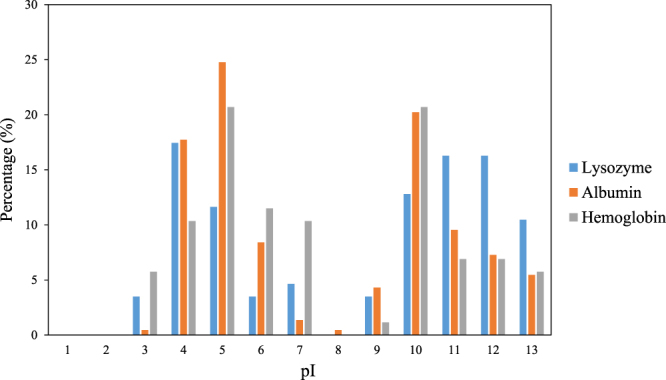
Percentage of ionizable amino acid residues with different pI values in the structure of the proteins.

Biomolecules used in this study include four amino acids with different functional groups and three proteins. The name and properties of amino acids and proteins used in this study are listed in Table 1. Because of their small size, as well as their simple chemical structure, the possible interactions between amino acids and C-S-H is anticipated to be primarily due to their functional groups. Charged amino acids included arginine and glutamic acid, the H-bonding amino acid was glutamine, and the hydrophobic amino acid was leucine. The three proteins studied were lysozyme, albumin, and hemoglobin. These proteins have been extensively studied for various applications including biomineralization of calcium carbonate^33^, synthesis of nanoparticles^34^, and synthesis of metal oxide nanocomposites and inorganic/organic hybrid materials^35–39^. The specifications of the proteins are given in Table 2. The isoelectric point (pI) value is estimated using H++ 3.0, an automated system that computes pI values of ionizable groups in biomolecules at the specified pH^40–43^. Albumin has the largest size among the proteins studied here. The percentage of amino acid residues with different pI values present in the structure of the proteins is shown in Fig. 1. It is evident that a higher number of amino acid residues with higher pI values are present in the structure of lysozyme, resulting in a higher pI value for this protein as seen in Table 2.

**Table 1:** List of biomolecules used in this study.

Name	Charge	Description
Arginine	Positive	Natural amino acid
Glutamine	Polar	Natural amino acid
Leucine	Nonpolar	Natural amino acid
Glutamic acid	Negative	Natural amino acid
Lysozyme	—	From chicken egg white
Albumin	—	Bovine serum albumin (BSA)
Hemoglobin	—	Human hemoglobin

**Table 2:** Specifications of the proteins used in this study.

Label	Lysozyme	Albumin	Hemoglobin
Molecular mass (g/mol)	14 k	65.5 k	64 k
Number of ionizable amino acid residues	86	440	87
pI	10.6	5.47	7.96

### Sample preparation

C-S-H in this study was synthesized in the form of powder using the direct precipitation method by the reaction of calcium and silicate ions in an aqueous solution^44^. C-S-H with varied initial C/S of 0.7 and 1.5 were prepared by mixing various amounts of CaCl_2_ and Na_2_SiO_3_ solutions. It should be noted that the C/S of the final products can slightly differ from that of the initial reactants^44^. Nonetheless, nominal C/S of 0.7 and 1.5 is used in this paper to refer to two C-S-H samples with distinct compositions. Amino acids with concentrations corresponding to 0.025 g of amino acid to 1 g of calcium salt, and proteins with a concentration corresponding to 0.3 g of protein to 1 g of calcium salt were added to the Na_2_SiO_3_ solution. The higher mass of proteins compared to amino acids was chosen because of the higher molar mass of the proteins compared to the amino acids. The 1 M CaCl_2_ solution was gradually added to the 0.22 M Na_2_SiO_3_ solution and stirred for seven days in a CO_2_ free glovebox to prevent carbonation. The solutions were filtered and washed with deionized water and acetone and then dried under vacuum for 3 days and then passed through a #60 sieve to obtain a fine powder. The fine powders were stored in the CO_2_ free glovebox and were only taken out when performing experiments to minimize the amount of exposure to air and carbonation. For nanoindentation testing, the powder was poured into a stainless steel mold and compacted using a loading machine to a pressure of 30 ksi to obtain a pellet with a diameter of 0.5 in. The pellets were cast in epoxy and then the surface of the samples was polished using SiC sand papers with grit numbers of 80, 180, 320, 600 and 1200, respectively. Isopropyl alcohol was used as a lubricating medium instead of water. Final polishing was performed using a diamond paste containing abrasive particles with a size of 1 μm. Samples were ultrasonically cleaned in isopropyl alcohol for 30 min to remove the polishing residues. The polished samples were used in performing nanoindentation. The sample powders for SEM, XRD, and FTIR were transferred in sealed glass vials.

### XRD analysis

The change in the interlayer distance of the control and modified C-S-H was examined using XRD. XRD is particularly effective in characterizing materials with layered structure and has been successfully utilized in studying cement-based materials^11,15,45^. The change in the interlayer distance of C-S-H was evaluated by measuring the change in the reflection peak corresponding to 002 basal spacing. The XRD scans were conducted using a Siemens X-ray diffractometer with the Cu Kα radiation at a scan rate of 0.3 degrees/min and 0.02 degrees/step.

### FTIR analysis

FTIR was used to study the polymerization of silicate chains in C-S-H nanocomposites. In this method, the characteristic stretching vibrations of (Si-O) bonds was used to identify different silicate bonds present in C-S-H^15,18,19^. The presence and intensity of the bridging silicate tetrahedra (Q^2^) and end silicate tetrahedra (Q^1^) can be detected using the FTIR spectra. The FTIR analysis was conducted using a Perkin Elmer Paragon 1000 FTIR equipped with an ATR accessory with a scan rate of 4 cm^−1^ in the range of 650 cm^−1^ to 4000 cm^−1^.

### Microscopy examination

Scanning electron microscopy (SEM) was utilized to investigate the effect of amino acids and proteins on the morphology of C-S-H nanocomposites. Powder samples were passed through the #200 sieve and placed on a carbon tape attached to a carbon stub. The surface of the samples was coated with a thin layer of gold or palladium to reduce the effect of charging for high magnification imaging. The SEM images were taken at an accelerating voltage of 20 KV using a PHILIPS XL30 ESEM-FEG device.

AFM imaging was conducted using a TT-AFM from AFMWorkshop. A silicon probe from AppNano with a cantilever length and width of 225 μm and 40 μm, a tip radius of less than 10 nm and spring constant of 36–90 N/m was used for imaging. Images of each sample were taken in the non-contact mode.

### Nanoindentation measurement

The Young’s modulus of the C-S-H samples was measured using AFM-based nanoindentation. AFM is suited to determine the Young’s modulus of materials with mechanical properties changing over nanoscales^10,46^ making it a promising method for the mechanical characterization of cementitious materials^10^. In an AFM-based indentation test, a probe with a known cantilever stiffness comes into contact with the material, indents up to a certain depth and then retracts from the surface. The deflection of the beam versus vertical position of the tip is recorded by the instrument. Deflection data is logged as an electrical signal in volts units that needs to be converted to distance units using a sensitivity factor. Sensitivity is calculated as the slope of a linear fit to voltage-distance curve when the same probe is pushed against a relatively hard surface compared to the tested material so that the indentation depth can be assumed to be negligible. Deflection is converted to force knowing the spring constant of the cantilever using Hooke’s law to obtain the force-distance curves. The amount of deflection corresponding to the cantilever response obtained from the test on a hard surface with no penetration is subtracted from the total deflection to generate the force-indentation distance curves.

The unloading portion of the load-indentation distance curve was fitted to the Hertz model using the following formula^47^ in a Matlab code:

1 $$F=\frac{4}{3}M{R}^{0.5}{\delta }^{1.5}$$

where *F* is the force in nN, *δ* is the indentation distance in nm, *R* is the tip radius in nm and *M* is calculated with the following expression:

2 $$\frac{1}{M}=\frac{1-{\nu }_{1}^{2}}{{E}_{1}}+\frac{1-{\nu }_{2}^{2}}{{E}_{2}}$$

where ν_1_ and *E*_1_ are the Poisson’s ratio and Young’s modulus (GPa) of the material and *ν*_2_ and *E*_2_ are the Poisson’s ratio and Young’s modulus (GPa) of the AFM tip. Since a diamond probe was used in this study and its Young’s modulus is much higher than that of the tested material, the above-mentioned expression was simplified as follows:

3 $$\frac{1}{M}=\frac{1-{\nu }_{1}^{2}}{{E}_{1}}$$

In this study a cube corner diamond probe (DNISP from Bruker) with a spring constant of 291 N/m and tip radius of 40 nm based on the specification provided by the manufacturer was used for nanoindentation. Nanoindentation tests were performed on about 48 points located in three random meshes of 5 µm spacing for each of the polished samples. The deflection sensitivity was calibrated using a sapphire substrate purchased from Bruker.

## Results and Discussion

### XRD results

**Figure 2:**
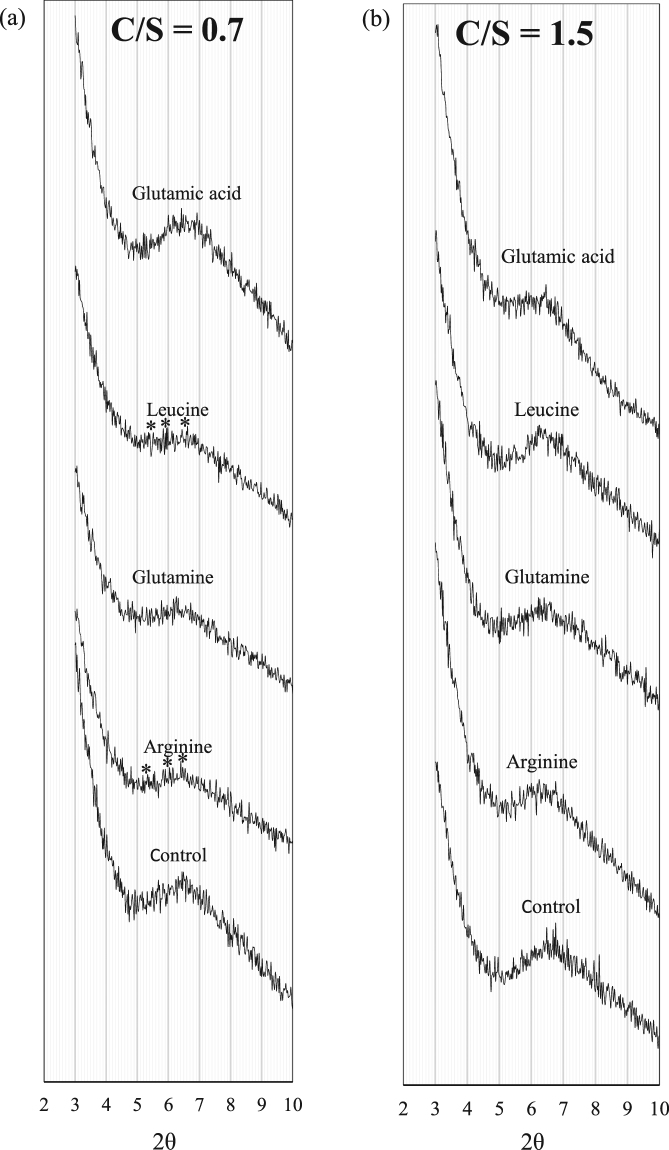
XRD spectra of C-S-H modified with amino acids at C/S of (**a**) 0.7 and (**b**) 1.5.

There are several studies on the stoichiometry of C-S-H, suggesting various models to describe its chemical structure^11,48,49^. It is generally acknowledged that C-S-H does not have a specific chemical formula and stoichiometry^13,17,20,50^. However, C-S-H stacks, with their limited lateral extension, follow an ordered structure in localized regions. In this type of structure, the volume fraction of interlayer distance is significantly lower than that of the pores between the C-S-H stacks referred to as gel pores^20^. The XRD spectra for the control C-S-H and C-S-H samples modified with amino acids at a C/S of 0.7 and 1.5 are shown in Fig. 2a,b, respectively. The 2θ range of 3° to 10° corresponds to the peak of the d_002_ basal spacing reflection. The interlayer distance for all the samples is calculated using Bragg’s equation^51^:

4 $$2dSin\theta =n\lambda $$

where *d* is the interlayer distance (Å), *θ* is the peak angle (degrees), n is 2 and *λ* is 1.5418 (Å). The calculated interlayer distance for different C-S-H samples is shown in Table 3. At C/S of 0.7, C-S-H samples modified with arginine (positively charged), leucine (non-polar) and glutamine (H-bond forming) show a broader and less intense peak compared to that of the control sample. In the spectra of arginine and leucine, multiple peaks (marked with * in Fig. 2a) are observed. There is no change in the intensity of the peak in the C-S-H sample modified with glutamic acid. The presence of other peaks at lower angles in addition to the main peak in the spectra of C-S-H samples modified with leucine and arginine can be attributed to a partial intercalation of amino acids between the C-S-H layers increasing the interlayer distance in some of the C-S-H stacks. It is noted that the interlayer distance has slightly increased in the C-S-H samples modified with glutamine which could be attributed to possible adsorption of glutamine in between C-S-H layers. It is seen that there is no indication of intercalation in the C-S-H samples modified with glutamic acid (negatively charged) as the interlayer distance has not increased compared to the control sample. This is in general agreement with the results of adsorption using phage display of peptides performed by Picker *et al*.^30^ on C-S-H sample with C/S of 0.66. Based on their results at low C/S, positively charged, polar (H-bond forming) and hydrophobic amino acids adsorb onto the C-S-H.

**Table 3:** The basal diffraction angle and interlayer distance detected in the XRD spectra of the control and modified C-S-H powders.

	C/S = 0.7		C/S = 1.5	
	2θ	d (Å)	2θ	d (Å)
Control	6.5	13.6	6.7	13.2
Arginine	6.4	13.8	6.2	14.3
Glutamine	6.3	14.1	6.4	13.8
Leucine	6.5	13.6	6.3	14.1
Glutamic acid	6.6	13.4	6.4	13.8
Lysozyme	6.4	13.8	6.2	14.3
Albumin	6.4	13.8	6.5	13.6
Hemoglobin	6.6	13.4	6.5	13.6

Their study showed that negatively charged amino acids have the lowest adsorption at this C/S ratio. The deprotonated silanol groups of C-S-H with negative surface charges favor the electrostatic interactions with the positively charged amino acid arginine, H-bond formation with the polar amino acid glutamine and van der Waals interaction with hydrophobic amino acids^30^.

The interlayer distance of the control C-S-H sample with C/S of 1.5 is lower than that of the control sample with C/S of 0.7. This increase in crystalline size along the c axis with decreasing C/S ratios is in agreement with previous studies^17,51^. At C/S of 1.5, the C-S-H samples modified with glutamine (H-bond forming) and glutamic acid (negatively charged) show broader peaks compared to that of the control sample. It is seen that the interlayer distance increased in the samples modified with arginine and leucine indicating the intercalation of amino acids in C-S-H. In the C-S-H sample modified with glutamine the interlayer distance has slightly increased compared to the control sample, indicating the potential intercalation of glutamine in the interlayer space of C-S-H. In the sample modified with glutamic acid another peak in addition to the main peak appeared at a lower angle. This could imply the intercalation of glutamic acid at this C/S ratio, compared to C/S of 0.7, where no such adsorption was observed. An explanation for the increased influence of negatively charged glutamic acid on the C-S-H structure can be sought in the structure of C-S-H. As mentioned previously at low C/S ratio of 0.7, the surface of C-S-H carried negative charges due to the deprotonation of silicate groups; the negatively charged glutamic acid is less likely to interact with C-S-H. With increasing C/S ratio to 1.5, the concentration of Ca^2+^ is increased and as a result, Ca^2+^ bonds to negatively charge surface of C-S-H and shields the surface against positively charged amino acids. In this case, the adsorption of negatively charged amino acid, glutamic acid, onto C-S-H surface is facilitated through bridging with Ca^2+^ ^30,31^.

**Figure 3:**
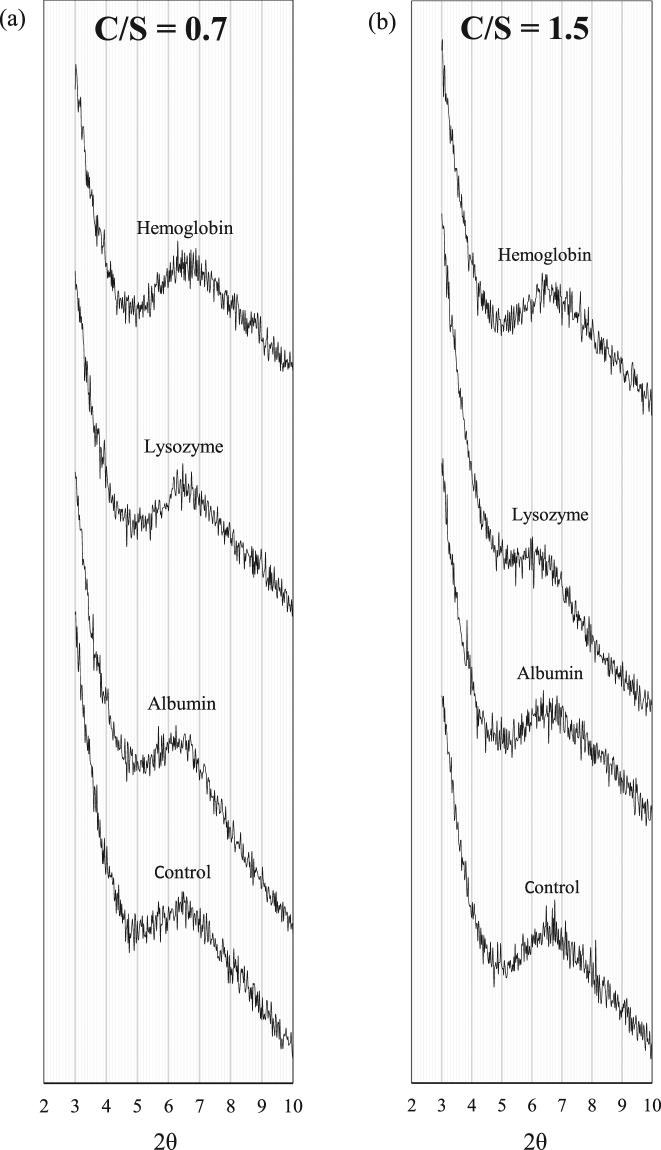
XRD spectra of C-S-H modified with the proteins at C/S of (**a**) 0.7 and (**b**) 1.5.

The XRD spectra of the control C-S-H and C-S-H samples modified with different proteins are shown in Fig. 3a,b. There is no significant change in the interlayer distance of the samples modified with proteins at C/S of 0.7. However, at C/S of 1.5, the peak corresponding to the C-S-H sample modified with lysozyme has broadened and shifted toward lower angles. As shown in Table 3, the interlayer distance of the C-S-H samples modified with lysozyme has increased compared to the control sample. There is not a significant change in intensity of the basal reflection peak and interlayer distance of the samples modified with albumin and hemoglobin. The larger size of proteins compared to amino acids could be a factor in the lower likelihood of intercalation of proteins compared to amino acids in C-S-H.

**Figure 4:**
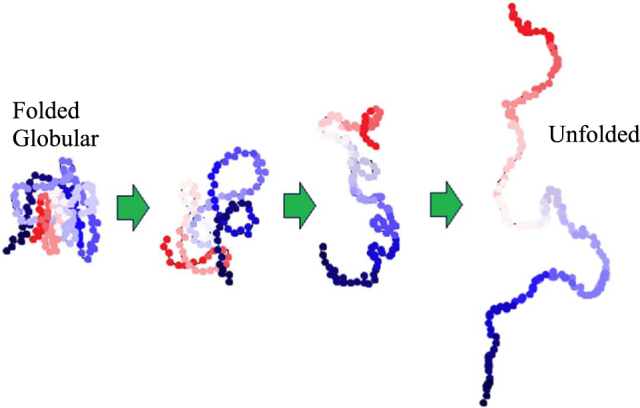
Schematic of how proteins undergo conformational changes with increasing pH. Different colors are used to indicate distinct functional groups in proteins.

The observed influence of lysozyme can be attributed to the specific conformation and composition of this protein. The pH value of the C-S-H solutions right after mixing was measured to be 11.0 and 10.0 at C/S of 0.7 and 1.5, respectively. The proteins at their stable and active state (called the native state) fold into the most thermodynamically favorable structure and tend to take a globular shape^52^. In this state, the hydrophobic residues are located in the interior of the protein molecules and the hydrophilic residues are exposed at the exterior of the protein molecule. The hydrophobic interaction at the interior and hydrophilic interaction at the exterior are both responsible for stabilizing the protein molecules at their native stage. The change in the pH interferes with the stabilizing interactions and forces the protein molecules to unfold^53–57^. A schematic of how the proteins unfold at high pH is shown in Fig. 4. This exposes various residues with different functionalities providing additional pathways for the interaction between the protein and C-S-H. The observed influence of lysozyme on the interlayer distance of C-S-H at C/S of 1.5 could be related to the specificity of this biomolecule favoring such effect on C-S-H. The specificity of biomolecules in their interaction with inorganic surfaces has been observed and studied in prior investigations^58,59^. Further investigations based on computational approaches can provide valuable insights into the effect of protein conformation on the atomic structure of C-S-H.

It should be emphasized here that some biomolecules could physically adsorb onto the starting inorganic chemicals used to synthesize C-S-H, and this results in a reduction in the target C/S of the produced C-S-H and consequently an increase in the interlayer distance. Further investigations are necessary to decouple such potential effect of biomolecules on the final C/S from the effect of biomolecules on the nanostructure of C-S-H.

### FTIR Analysis

**Figure 5:**
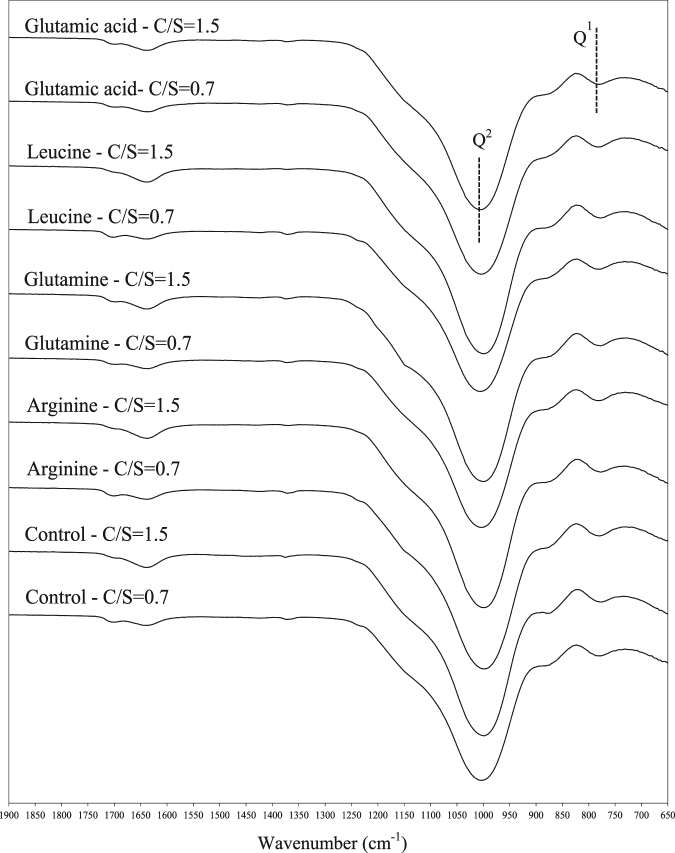
FTIR spectra of C-S-H modified with amino acids at C/S of 0.7 and 1.5.

**Figure 6:**
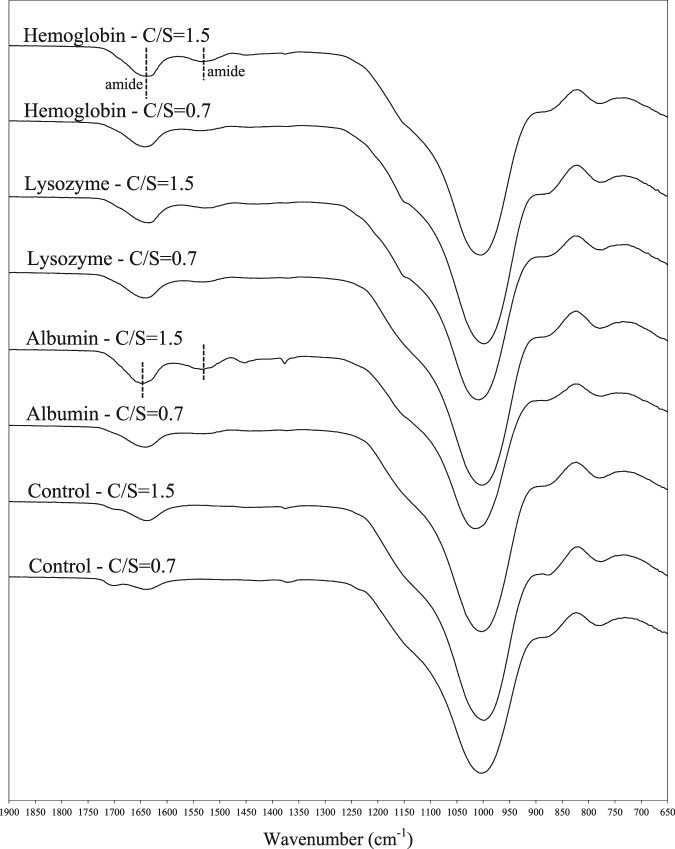
FTIR spectra of C-S-H modified with proteins at C/S of 0.7 and 1.5.

The FTIR spectra for the control C-S-H and C-S-H samples modified with amino acids and proteins at C/S of 0.7 and 1.5 are shown in Figs 5 and 6, respectively. The frequencies of the Si-O stretching vibrations of the Q^1^ and Q^2^ tetrahedra for different C-S-H samples are listed in Table 4. Q^1^ represents the end silicate tetrahedra and Q^2^ corresponds to the bridging silicate tetrahedra. The intensity ratio of Q^2^ to Q^1^ is a measure of polymerization of silicate chains in C-S-H and is shown in Table 4.

**Table 4:** The wavelength numbers corresponding to Q^2^ and Q^1^ silicates in the FTIR spectra of the control and modified C-S-H powders.

	C/S = 0.7			C/S = 1.5		
	Q^2^ (cm^−1^)	Q^1^ (cm^−1^)	Q^2^/Q^1^	Q^2^ (cm^−1^)	Q^1^ (cm^−1^)	Q^2^/Q^1^
Control	1004	778	3.80	999	777.5	3.44
Arginine	999.5	779.5	3.55	999.5	777	3.51
Glutamine	1003	782.5	3.77	999.5	777.5	3.42
Leucine	1004	781	3.85	999.5	777.5	3.41
Glutamic acid	1003.5	781.5	3.57	1004	780	3.46
Lysozyme	1003	779	3.59	1008	775	3.62
Albumin	1003	779	3.70	1015.5	775.5	3.68
Hemoglobin	999	779	3.41	1004	776.5	3.44

It is observed that the location of the Q^2^ band is shifted towards higher frequencies in the control sample with a reduction in C/S ratio. This is an indication of increased polymerization in the control sample with a C/S of 0.7 compared to the sample with C/S of 1.5 as it is also evident from the higher intensity ratio of Q^2^ to Q^1^ at the lower C/S^15,60^. It is seen that the location of the Q^2^ band in arginine sample at C/S of 0.7 is lower than that of the control C-S-H, suggesting a reduction in polymerization as a result of interaction between arginine and C-S-H at this C/S ratio. The intensity ratio of Q^2^/Q^1^ in the sample modified with arginine is also lower than that of the control C-S-H. The Q^2^ peak frequency of the C-S-H with other amino acids except arginine is seen to be unchanged compared to the control C-S-H at C/S of 0.7. At C/S of 1.5, the Q^2^ peak of arginine shows a similar frequency to the control C-S-H. It is interesting to note the shift to higher frequencies of Q^2^ in the C-S-H modified with glutamic acid at this C/S ratio, indicating the interaction of glutamic acid with C-S-H and maybe an indication of increased polymerization in C-S-H modified with glutamic acid; however, the intensity ratio Q^2^/Q^1^ appears to remain unchanged in this sample. The reduction in the influence of arginine and an increase in influence of glutamic acid with increasing C/S ratio is consistent with the XRD results discussed earlier.

In the samples modified with proteins, at C/S of 0.7 there is a shift to lower frequencies in the Q^2^ peak of the C-S-H modified with hemoglobin. The corresponding Q^2^ peak frequency of the C-S-H modified with albumin and lysozyme is unchanged. With increasing C/S ratio from 0.7 to 1.5 the Si-O stretching band corresponding to Q^2^ shifted to higher frequencies in C-S-H samples modified with proteins. This trend is not similar to that of the control C-S-H sample and C-S-H modified with amino acids. One explanation would be the calcium binding effect of proteins. The proteins can bind the added calcium ions so the actual C/S ratio is lower than what is expected^61^. To investigate the calcium binding effect of proteins, conductivity measurement was utilized. The binding between proteins and Ca^2+^ reduces the concentration of free Ca^2+^ in the solution, which results in a reduction in the conductivity of the solutions. About 100 mL of 0.002 M Ca(OH)_2_ solutions without proteins and with 0.12 g of proteins were prepared and their conductivity was measured. Two electrodes consisting of stainless steel rods with a diameter of 6 mm were inserted inside a glass beaker with a dimeter of 70 mm. The conductivity of the solutions was measured using a Reference Gamry 600 potentiostat/galvanostat with a 20 mV AC signal and a frequency range of 10^6^ to 10 Hz. The normalized conductivity of the solutions with proteins per the conductivity of the solution without proteins after one hour and after 48 hours of mixing is shown in Table 5. It is seen that the solutions with proteins showed a lower conductivity compared to the solution without proteins, indicating possible binding of proteins to Ca^2+^.

**Table 5:** The normalized conductivity of the Ca(OH)_2_ solutions with proteins per the conductivity of the Ca(OH)_2_ solution without proteins, after one hour and after 48 hours of mixing.

	Lysozyme	Albumin	Hemoglobin
After 1 hour	0.60	0.64	0.62
After 48 hours	0.46	0.48	0.45

The second explanation for the notable shift in the Q^2^ frequencies of C-S-H with all proteins at C/S of 1.5 could be the incomplete nucleation of C-S-H particles at C/S of 1.5 due to the presence of proteins. Consequently, the solution has a high content of unreacted silicate gel and surface adsorbed proteins^31^. The presence of the bands corresponding to amide I and amide II at around 1640 cm^−1^ and 1530 cm^−1^, respectively, indicates the high content of proteins in the C-S-H samples modified with the proteins at C/S ratio of 1.5^62,63^.

The shift in the Q^2^ frequencies of C-S-H with all proteins can also be viewed as an indication of increased polymerization in the C-S-H samples and suggests the influence of the proteins on silicate polymerization of C-S-H. While the influence of lysozyme on C-S-H is also observed from the XRD results, such an influence of albumin and hemoglobin on C-S-H structure is not evident from the XRD analysis.

### Morphology examination

**Figure 7:**
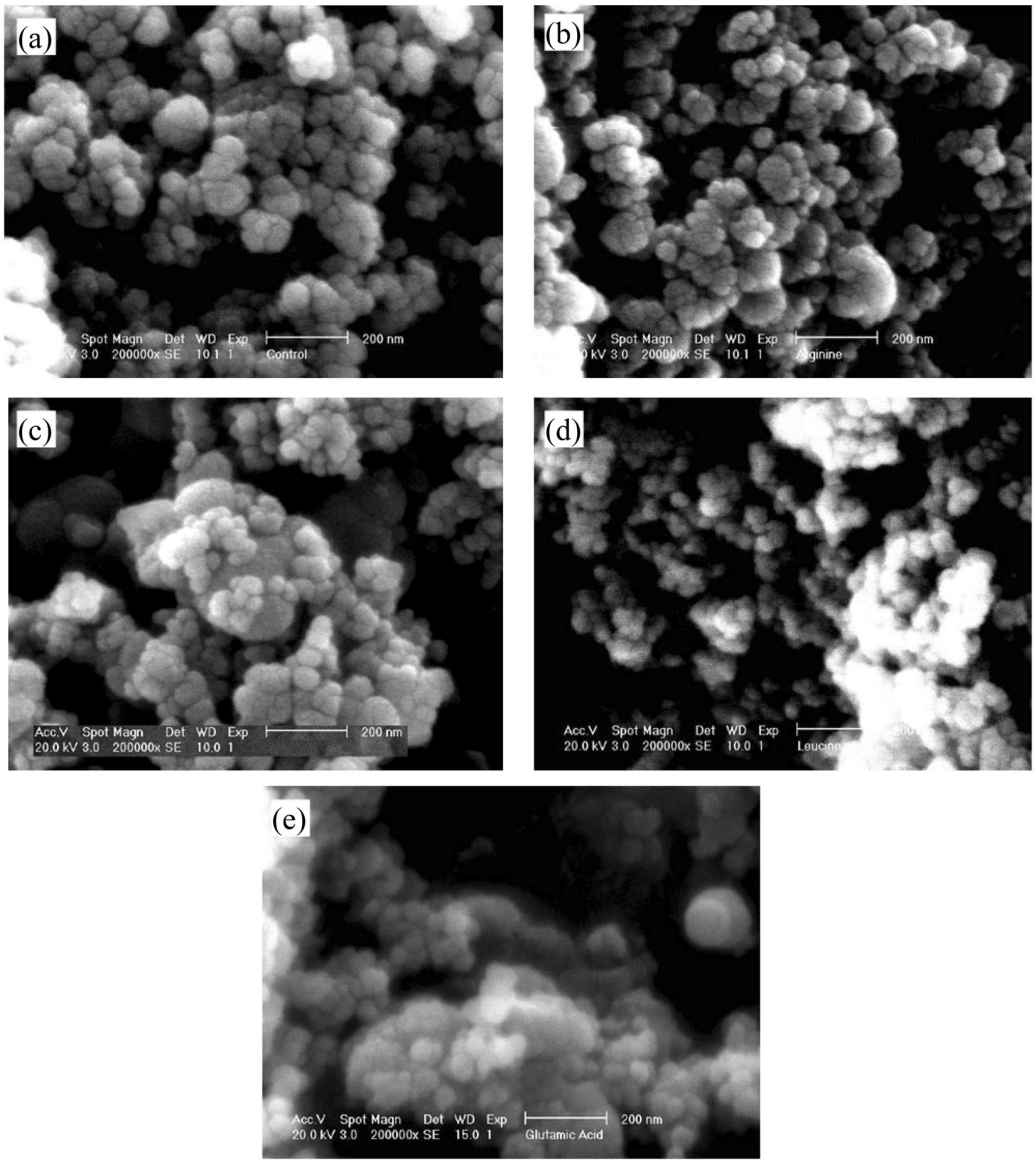
The morphology of the (**a**) control and modified C-S-H with (**b**) arginine, (**c**) glutamine, (**d**) leucine, and (**e**) glutamic acid at C/S of 0.7.

The SEM images of the control C-S-H and C-S-H samples modified with arginine, glutamine, leucine and glutamic acid at C/S of 0.7 are shown in Fig. 7. There is no noticeable difference in the morphology of the control sample and the C-S-H samples modified with amino acids as seen from SEM images. The globular morphology with a size of 20 nm to 100 nm can be observed in all C-S-H samples, which is in agreement with the previous studies^11,64^.

**Figure 8:**
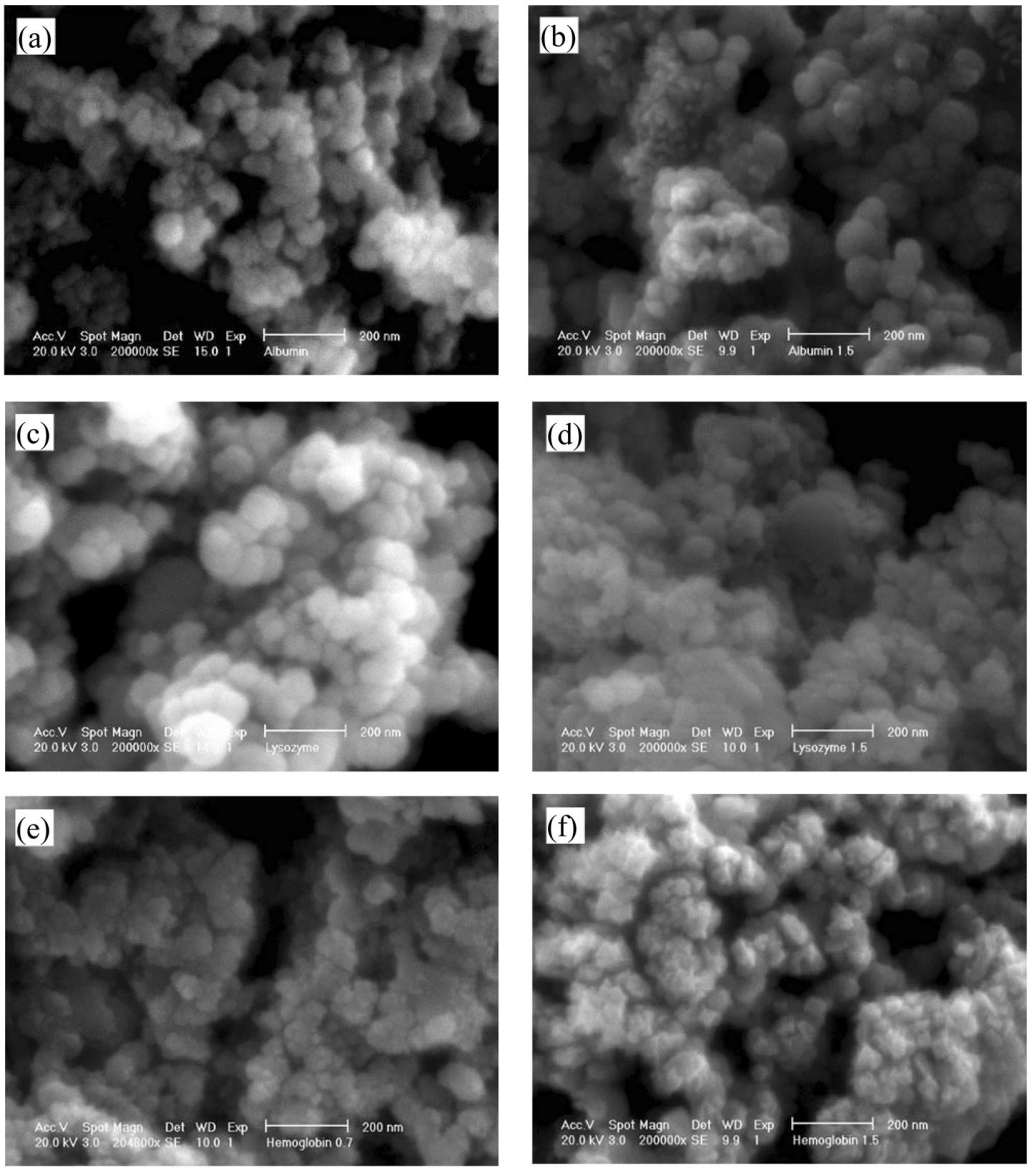
The morphology of the modified C-S-H with (**a**) albumin at C/S of 0.7, (**b**) albumin at C/S of 1.5, (**c**) lysozyme at C/S of 0.7, (**d**) lysozyme at C/S of 1.5, (**e**) hemoglobin at C/S of 0.7 and (**f**) hemoglobin at C/S of 1.5.

The SEM images of the C-S-H samples modified with albumin, lysozyme and hemoglobin at C/S of 0.7 and 1.5 are shown in Fig. 8. The morphology of the albumin and lysozyme modified C-S-H is comparable to the control sample. The C-S-H sample modified with hemoglobin at both C/S ratios showed a slightly different morphology. The C-S-H particles in these samples are observed to exhibit a platelet morphology and are less round compared to other C-S-H samples. In this structure, the network is built of pores and building blocks of nano-platelets. This type of structure has been previously observed by researchers in hierarchically nanostructured mesoporous spheres of C-S-H^65^.

**Figure 9:**
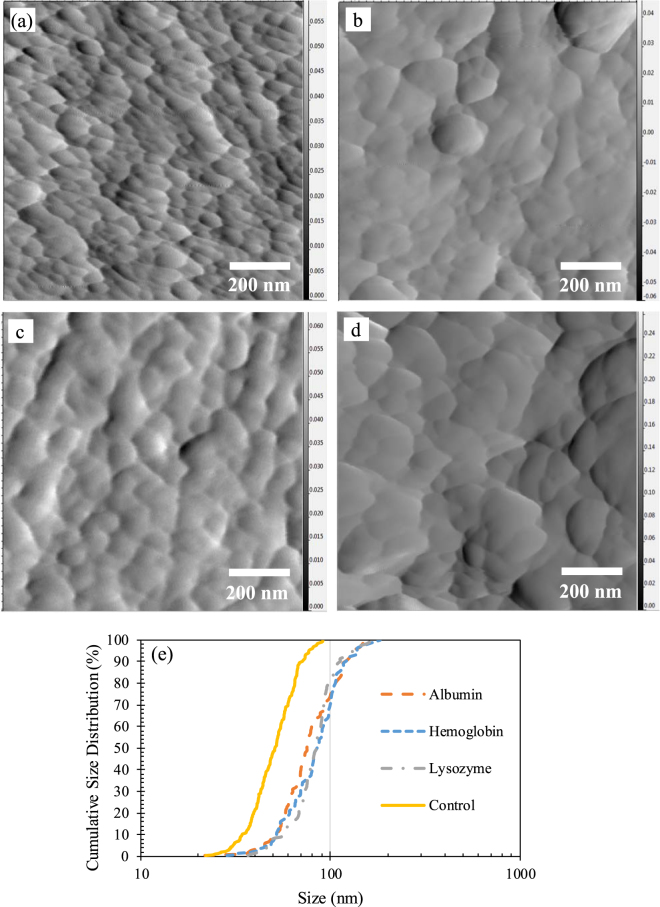
Amplitude error image of (**a**) the control C-S-H sample and C-S-H samples modified with (**b**) albumin (**c**) lysozyme and (**d**) hemoglobin at C/S of 1.5. (**e**) Cumulative size distribution of the nanoparticles shown in Figures (**a**–**d**).

AFM imaging was also utilized to provide more detail regarding the morphological attributes of the C-S-H microstructure. The amplitude error AFM images of the control C-S-H and the C-S-H samples modified with albumin, lysozyme and hemoglobin at C/S of 1.5 are shown in Fig. 9. The amplitude error images were chosen because the boundaries of the nanoparticles can be more readily distinguished in this type of image. The cumulative size distribution of the nanoparticles was measured using the ImageJ software and is shown in Fig. 9e. The size of a nanoparticle is assumed to be equal to the diameter of a circle with the equal area as the nanoparticle. It is seen that the nanoparticles seem to be larger in C-S-H modified with proteins compared to the control C-S-H. The increase in the size of C-S-H nanoparticles modified with proteins can be related to the templating effect of proteins directing the aggregation of C-S-H after nucleation and during growth leading to an increase in the size of the nanoparticles. It is interesting to note the increase in the size of the C-S-H nanoparticles modified with the proteins in light of increase in silicate polymerization at C/S ratio of 1.5 as seen from Table 4. More detailed studies are needed to fully elucidate the relation between morphology and silicate polymerization in the modified C-S-H.

### Mechanical property

**Figure 10:**
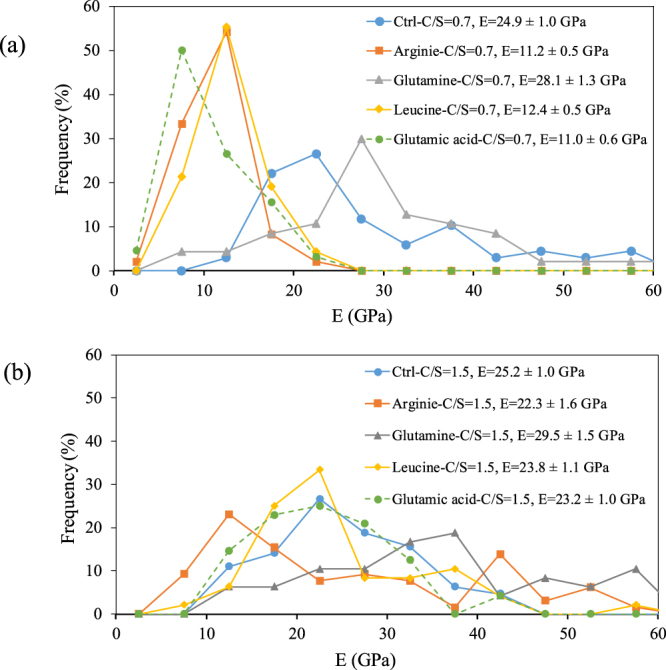
Elastic modulus values of the control C-S-H and C-S-H samples modified with amino acids at (**a**) C/S of 0.7 and (**b**) C/S of 1.5.

The Elastic modulus measurements of the control C-S-H and C-S-H samples modified with amino acids at C/S of 0.7 and 1.5 are shown in Fig. 10. Comparing the elastic modulus value of the control samples with C/S of 0.7 and 1.5, it is noted that no significant difference can be observed. Excluding the values more than 45 GPa, which could be attributed to calcium hydroxide^66^, an average Young’s modulus of 24.9 ± 1.0 GPa for the control C-S-H with C/S of 0.7 and 25.2 ± 1.0 GPa for the control C-S-H samples with C/S of 1.5 was obtained. These values are consistent with the values obtained in previous studies^66–68^. It was shown by Beaudoin and Feldman^69^ that the intrinsic elastic properties of C-S-H are not dependent on the C/S ratio. This indicates that the decrease in the Young’s modulus of C-S-H, observed in nanoindentation test results, could be because of a decrease in the packing density of C-S-H^67^.

At C/S of 0.7, the C-S-H modified with arginine, leucine and glutamic acid showed lower values of Young’s modulus compared to that of the control sample. The decrease in the value of Young’s modulus may be due to the presence of these amino acids adsorbed on the surface or in the voids between the C-S-H particles resulting in a lower packing of C-S-H sample compared to the control C-S-H sample^18,19^. However, no noticeable change was observed in the sample modified with glutamine. At C/S of 1.5, all the samples modified with amino acids have average values comparable to that of the control sample. However, the arginine sample has its higher intensity peak at lower values compared to the other samples potentially related to a reduction in the packing density in C-S-H modified with arginine.

**Figure 11:**
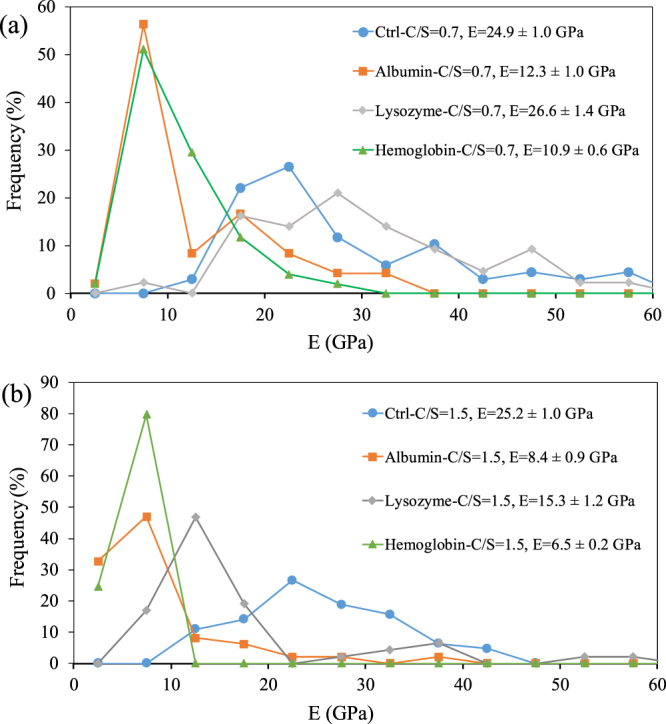
Elastic modulus values of the control C-S-H and C-S-H samples modified with proteins at (**a**) C/S of 0.7 and (**b**) C/S of 1.5.

The elastic modulus values of the control C-S-H and C-S-H samples modified with proteins at C/S of 0.7 and 1.5 are shown in Fig. 11. At C/S of 0.7, the C-S-H modified with lysozyme is comparable to the control sample. However, albumin and hemoglobin showed lower values of Young’s modulus compared to that of the control sample.

At C/S of 1.5, all the C-S-H samples modified with proteins had lower values of Young’s modulus compared to that of the control sample. In addition, the Young’s modulus of C-S-H with proteins seems to be slightly lower at C/S = 1.5 than at C/S = 0.7. This can be related to a change in silicate polymerization of C-S-H, as seen from the FTIR results shown in Fig. 6, as well as a change at the mesoscale in terms of increased occupation of gel pores by the proteins thereby reducing packing density of C-S-H. It is noted that the sample modified with lysozyme has higher values of Young’s modulus compared to samples modified with albumin and hemoglobin at C/S = 1.5.

## Conclusions

In this study the effect of biomolecules with distinct functional groups and various molecular sizes on the atomic structure, morphology and Young’s modulus of C-S-H was investigated. The findings of this study are as follows:

- The XRD analysis showed a change in the atomic structure, in the form of intercalation or surface adsorption, of C-S-H modified with the H-bond forming, hydrophobic and positively charged amino acids at both C/S = 0.7 and 1.5. With an increase in C/S ratio, the influence of negatively charged amino acid became more pronounced. Among the proteins studied here, lysozyme appeared to show intercalation in C-S-H only at high C/S = 1.5. This could be related to the composition and conformation of this protein facilitating intercalation of this protein in C-S-H at this C/S.
- The FTIR results indicated the effect of arginine on the silicate polymerization of C-S-H at low C/S ratio and increased influence of glutamic acid at higher C/S = 1.5. A noticeable increase in the silicate polymerization of samples modified with proteins at C/S = 1.5 was observed.
- The control C-S-H and the C-S-H modified with amino acids used in this study demonstrated a globular morphology of C-S-H nanoparticles. C-S-H modified with lysozyme and albumin also showed a globular morphology. The C-S-H samples modified with hemoglobin showed a platelet morphology in contrast to a globular morphology of the C-S-H modified with other proteins.
- A decrease in the Young’s modulus of the C-S-H samples modified with amino acid was observed at lower C/S. However, the effect of amino acids in altering the Young’s modulus of C-S-H system was not significant at high C/S.
- The Young’s modulus of C-S-H with proteins was lower than that of the control C-S-H at both C/S values. The reduction in the Young’s modulus was slightly increased at high C/S ratio. C-S-H modified with lysozyme was shown to exhibit a higher Young’s modulus than C-S-H modified with albumin and hemoglobin.
